# Energy Management in Smart Cities Based on Internet of Things: Peak Demand Reduction and Energy Savings

**DOI:** 10.3390/s17122812

**Published:** 2017-12-05

**Authors:** Chinmaya Mahapatra, Akshaya Kumar Moharana, Victor C. M. Leung

**Affiliations:** 1 Department of Electrical and Computer Engineering, The University of British Columbia (UBC), 2332 Main Mall, Vancouver, BC V6T 1Z4, Canada; vleung@ece.ubc.ca; 2 Power Systems Studies, Powertech Labs Inc., Surrey, BC V3W 7R7, Canada; akshaya.moharana@powertechlabs.com

**Keywords:** information and communication technologies, smart cities, smart home, home energy management, *Q*-learning, user convenience, peak demand, carbon footprint

## Abstract

Around the globe, innovation with integrating information and communication technologies (ICT) with physical infrastructure is a top priority for governments in pursuing smart, green living to improve energy efficiency, protect the environment, improve the quality of life, and bolster economy competitiveness. Cities today faces multifarious challenges, among which energy efficiency of homes and residential dwellings is a key requirement. Achieving it successfully with the help of intelligent sensors and contextual systems would help build smart cities of the future. In a Smart home environment Home Energy Management plays a critical role in finding a suitable and reliable solution to curtail the peak demand and achieve energy conservation. In this paper, a new method named as Home Energy Management as a Service (HEMaaS) is proposed which is based on neural network based *Q*-learning algorithm. Although several attempts have been made in the past to address similar problems, the models developed do not cater to maximize the user convenience and robustness of the system. In this paper, authors have proposed an advanced Neural Fitted *Q*-learning method which is self-learning and adaptive. The proposed method provides an agile, flexible and energy efficient decision making system for home energy management. A typical Canadian residential dwelling model has been used in this paper to test the proposed method. Based on analysis, it was found that the proposed method offers a fast and viable solution to reduce the demand and conserve energy during peak period. It also helps reducing the carbon footprint of residential dwellings. Once adopted, city blocks with significant residential dwellings can significantly reduce the total energy consumption by reducing or shifting their energy demand during peak period. This would definitely help local power distribution companies to optimize their resources and keep the tariff low due to curtailment of peak demand.

## 1. Introduction

Smart cities in brief can be defined as a city which uses information and communication technologies (ICT) such as smart sensors, cognitive learning, and context awareness to make lives more comfortable, efficient, and sustainable [1]. Cities today face multifarious challenges, including environmental sustainability, low carbon solutions and providing better services to their citizens. Given these trends, it is critical to understand how ICT can help make future cities more sustainable. As microcosms of the smart cities, smart and green buildings and homes stand to benefit the most from connecting people, process, data, and things. The Internet of Things (IoT) is a key enabler for smart cities, in which sensing devices and actuators are major components along with communication and network devices. Management of smart homes often requires analyzing IoT data from the interconnected networked devices to optimize efficiency, comfort, safety, and to make decisions faster and more precise [2]. Internet of Things (IoT) is a decade-old term for the interconnection of a plethora of heterogeneous objects and things over a global network so that they can exchange data and interact in real-time. Technologies, such as radio frequency identification, wireless sensor networks, artificial intelligence and machine learning, form the backbone of such interactions. The telecommunications sector estimates that by 2020 more than a half billion devices will be connected with each other [3].

The significant efficiency gains from home automation can make cities sustainable in terms of resources. Importantly, the IoT ambitions and scope are designed to respond to the need for real-time, context-specific information intelligence and analytics to address specific local imperatives [4]. Further, realization of smart, energy-efficient and green home infrastructure would allow the development of “livable” interconnected communities, which will form the backbone of a futuristic green city architecture [5]. Hence, energy management in smart homes is a key aspect of building efficient smart cities [6]. Energy management consists of demand side management (dsm), peak load reduction and reducing carbon emissions [7]. In an industrialized country, residential and commercial loads in urban centers consume a significant amount of electrical energy. As per the survey report [8] nearly 39–40% of the total energy consumption in Canada is consumed by the residential and commercial complexes. It is evident from various load surveys that the demand of electricity in these residences is highly variable and changes throughout the day. Therefore, finding suitable strategies for efficient management of home energy demand and to help reduce the energy consumption during peak period will make the communities’ more energy efficient. The Canada Green Building Council is working towards finding ways of making buildings greener and community sustainable [9]. Therefore, the need for energy efficient buildings is growing rapidly.

The power systems require equilibrium between electricity generation and demand [10]. Power system operators dispatch generating units primarily based on operating cost or market bid price. In order to meet the increased demand during peak period, more resources are often required to increase the generation capacity. Since addition of resources to meet the peak demand is an expensive investment, distribution system planners and utility engineers very often consider the reduction in peak load as a feasible solution to the problem. However, peak load reduction is mostly valuable for utilities and most popular only in a purely market-driven energy management environment. Under these circumstances, Demand Response (DR) [11,12] offers an opportunity for consumers to play a significant role in the operation of the electric grid by reducing or shifting their electricity consumption during peak periods in response to time-based rates or other forms of financial incentives. In most of the cases, DR is a voluntary program that compensates the consumers. There are many modern methods that reduce the peak load and load at peak time which is referred as Demand Side Management (DSM) [13]. Current market framework and lack of experience and understanding of the nature of demand response are the most common challenges in DSM nowadays [13].

Newer technologies like energy management using smart meters are now becoming popular in places like Ontario, Canada where few utilities have introduced energy tariff based on the Time-Of-Use (TOU) model in which a consumer pays differently for the energy consumption at the different time of the day. This has been possible due to the implementation of smart meters which track the energy usage in a home on an hourly basis [14] and then consumption information is bundled into multiple TOU price brackets. However, all these processes mostly help the local distribution company and in order to take advantages of the TOU, each household has to adopt a change in the use of the appliances which may cause significant discomfort to the consumers. In this scenario home appliance scheduling with electrical energy services for residential consumers is useful.

Recent developments in the area of information and communication technologies have provided an advanced technical foundation and reliable infrastructures for the smart house with a home energy management system [15,16]. Development of low power, cost-efficient and high performance smart sensor technologies have provided us with the tools to build smart homes [17,18]. As a result, a service platform can be implemented in a smart home to control the DR intelligently. This type of system should also give the users enough flexibility to input their choices while deciding on control of home devices [19] This makes the system more coherent, user friendly and scalable. In this paper, a home energy management system named as Home Energy Management as a Service (HEMaaS) is proposed which provides intelligent decisions, is interactive with the environment, scalable and user friendly. Wi-Fi connected smart sensors with centralised decision-making mechanism can identify peak load conditions and employ the automatic switching to divert or reduce power demand during peak period, thereby reducing the energy consumption. While different hardware, software, communication architectures have been proposed and compared by their power consumption, performance, etc. [20,21,22], the cost of implementing the infrastructure like: hardware devices, software framework, communication interfaces, etc. are still high enough that hinder the process of implementing the smart home technology for ordinary users. Moreover, the hardware and software architectures may not be able to handle the growing number of sensors and actuators with their heterogeneity. Therefore, by implementing monitoring and controlling sections of the HEMaaS platform using web services, one may achieve the agility, flexibility, scalability, and other features required for a feasible and affordable HEMaaS platform.

In this paper, peak reduction DR problem is formulated based on an agent-learning framework. Many authors have attempted to address this problem using multiple tools such as model predictive control [23], particle swarm optimization [24], iterative dynamic programming-based [25] and gradient-based methods [26]. However, these models are probabilistic and do not constitute learning from interaction with the environment. Further, these models are mostly price based, where cost saving instead of user preferences is a predominant factor. Some other solutions proposed in [27,28] do consider *Q*-learning based agent interaction system, however they target only particular appliances like air conditioners and LED lights.

In [29], authors have proposed a fully-automated energy management system based on the classical *Q*-learning based Reinforcement Learning (RL). The modelling is delay based, where users have a way of inputting their energy requests via time-scheduling and the agent learns gradually with time to find the optimal solution. However, this approach has several limitations. The author assumes mathematical disutility fuction and consumer initiated energy usage. Finding disutility function for each home or residence is costly and difficult and too much user interaction is not desired for a interoperable energy management system. Reference [30] focuses on applying a batch RL algorithm to control a cluster of electric water heaters. A more relevant work is reported in [31], which proposes device-based Markov Decision Process (MDP) models. It assumes that the user behaviour and grid control signals are known. However, these assumptions are not realistic in practice as described in this paper. This paper uses neural networks to learns this behaviour from historical data. In [32], authors use a discrete-time MDP based framework to facilitate the use of adaptive strategies to control a population of heterogenous thermostatically controlled loads to provide DR services to the power grid using *Q*-learning. Again the application here is specific to load controlled by ambient temperature.

In this paper, authors have used a typical canadian residential apartment to investigate the effectiveness of the proposed home energy management service. The main objective of HEMaaS is to shift and curtail household appliance usages so the peak demand and total energy consumption can be reduced. A new neural network based reinforcement learning algorithm has been proposed in this paper to achieve the objectives. The classical *Q*-learning problem of the reinforcement learning has been formulated as a neural fitted supervised learning problem here and is named Neural Fitted *Q*-based Home Energy Management (*NFQbHEM*) algorithm. This paper designs a node-red framework based user interface for controlling home appliance action based on *NFQbHEM* algorithm. The reward matrix incorporates user convenience parameters for state-action transition and includes user preference, power cost savings, robustness measure and user input preferences to initialize the algorithm. Peak demand reduction is of major goal of this paper maximizing user convenience. In summary the contributions of the paper are as follows:

- **User interface**: Using a node-red development framework [33] (Node-RED is a web-based programming tool for wiring together hardware devices, APIs and online services.) and message queue telemetry protocol secure broker, a user interface has been designed. It incorporates intelligent energy management capability and provides user input options. Temperature control of appliances, operation rescheduling and *On/Off* commands are initiated through the interface.
- **Peak demand reduction**: Using the proposed HEMaaS methodology, a reward matrix is generated for each peak reduction threshold. There are four peak reduction thresholds considered in this paper: 5%,10%,15% and 20%. Based on the user convenience suitable load reduction decisions are obtained.
- **Fault tolerance and user privacy**: Taking different random combinations of robustness measure, it has been shown how the user convenience is affected when user privacy is compromised and system has hardware fault. This part of the results is specific to this paper and not shown anywhere in state-of-art literature.
- **Energy saving and Carbon-footprint reduction**: The energy savings and carbon emmission reduction has been shown for a community of 85 houses over a year.

The paper uses several abbreviations, which are defined in Table 1. This paper is organized as follows: Section 2 describes the HEMaaS platform and its architecture. Section 3 formulates the home energy management problem as a markov problem and its possible solution strategy is described using various modelling parameters. The *NFQbHEM* algorithm is explained in Section 4. The experimental results are shown in Section 5 for different cases. Finally, the paper is concluded in Section 6 followed by references.

## 2. Home Energy Management as a Service

Home energy management is a service platform for the users to efficiently perform demand side management and control. It consists of home appliances connected through a grid of interconnected network of devices with preference given to the user convenience. The platform may be used for different types of community houses (condo and town homes) to manage their energy consumption. The systems may be categorized into hardware and software architectures.

### 2.1. The Hardware Architecture

A typical home consists of various appliances. These appliances establish a connection with the user and provide them with the monitoring and controlling capabilities. They are to be monitored and controlled locally or remotely by a HEMaaS platform using a Sonoff wireless switch [34] (The Sonoff is a device that is to be put in series with the power lines allowing it to turn any device on and off remotely. Its voltage range is 90–250 V and it can handle a max current of 10 A). Most of the common home devices fall within the (current, voltage) range of Sonoff currently commercially available in the market.

**Figure 1:**
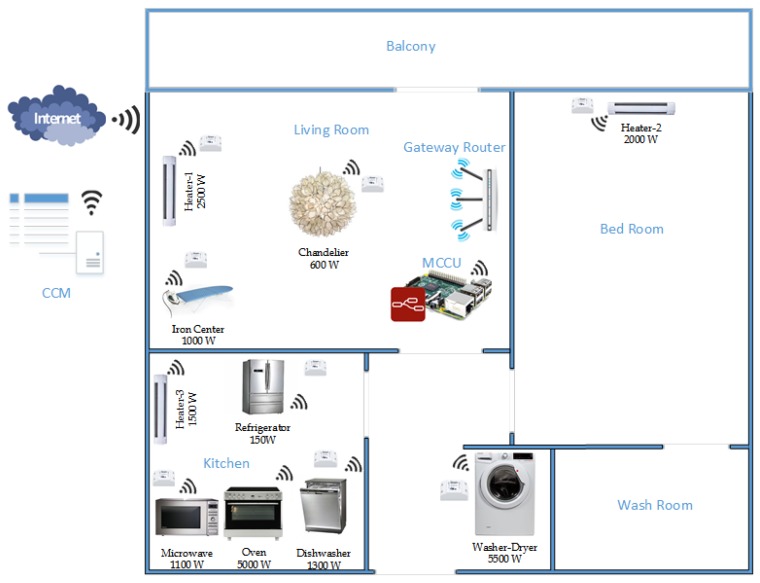
HEMaas hardware architecture of a typical Canadian condo.

The architectural diagram is shown in Figure 1. It consists of a Main Command and Control Unit (MCCU), Sonoff wireless switch, Smart meter, Gateway router and a Community Cloud Management panel (CCM). The MCCU is the main intelligence of the network which is responsible for triggering grid signals based on the output of the machine learning algorithms. It also has an input port which monitors for user input signals and accordingly provides user input to the controller. Sonoff Switch receives the trigger at its input port from the MCCU and turns the appliance Off/On accordingly. Smart meter provides power consumption data to the power station for overall efficient community energy management. Gateway router translates the MCCU messages using network address translation (NAT) in order to translate from a private network address (like 192.168.x.x, 10.0.x.x) to a public facing one. The smart meter and CCM are outside of gateway router and are separated by a secured firewall. CCM is monitored by the city power substation. The substation according to its generation and distribution has a set amount of available power for the community to use. CCM receives input from the substation and sends those commands to the each home’s MCCU which in term updates its power management strategy.

### 2.2. The Software Architecture and Communication Interface

**Figure 2:**
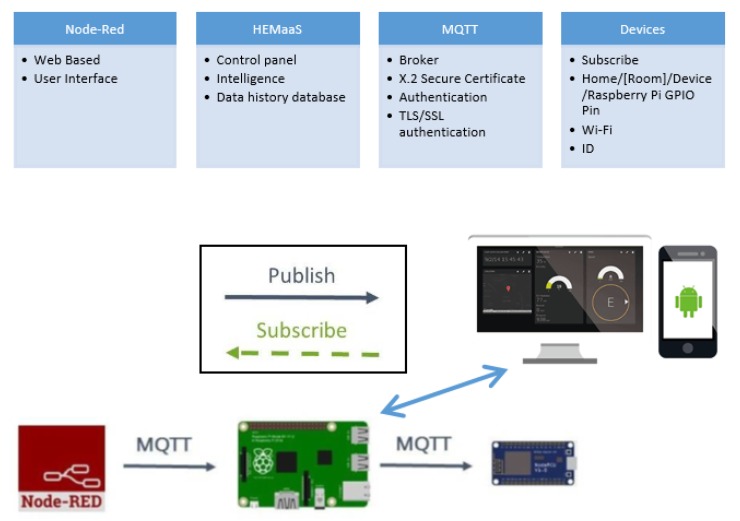
Software architecture and communication framework of HEMaaS platform.

The HEM MCCU needs to process the *NFQbHEM* algorithm integrating historical data as well as the user input preferences. Thus a decision needs to be formed quickly. Moreover, the state-action pair and user preferences change rapidly throughout the day and HEMaaS platform needs to provide service in a timely manner. Therefore, in this paper a Linux-based fast microcontroller has been used, namely Raspberry Pi3 [35]. Raspberry Pi3 runs the *NFQbHEM* algorithm using python programming language and plots the charts with its matplot library. Figure 2 shows the software architecture and communication framework of HEMaaS platform.

The web-based node-red programming model have been choosen to implement the controlling structure of the HEMaaS platform. It is easy to implement with a flow and is easily explandable if more appliances join the network. The user input is modelled inside the flow with a switch. User input manually can cause either a delay in the operation of the appliance or it will reset its temperature. These settings can also be changed via the smart MCCU algorithmic decision. A lightweight, low-power and secure protocol has been used in the paper to communicate between home appliances and the MCCU over Wi-Fi. The protocol is called Message Queue Telemetry Transport (MQTT) [36] and it is optimized for high-latency or unreliable networks. MQTT provides three level security for the data over the network. It uses a broker to publish messages to clients who subscribe to a particular topic. Topic are in the form of a hierarchy of devices in the home [Home/(Room)/(Device)/RaspberryPi GPIO Pin]. Mosquitto [37] broker has been used in this architecture. Broker performs authentication via username and password, client ID and X.2 certification to validate the clients in the HEM network. Thus intrusion can be prevented. A dashboard user interface (UI) for desktop and mobile have been designed to give users ample interaction opportunities. The design of the UI is described in detail in the result section.

## 3. HEM as a Markov Decision Process and Its Solution

We formulate our HEM problem as a set of discrete states, where each state represents a binary formulation of the power levels of home appliances. The MCCU issues command to switch these power states. We model the power states as a Markov Decision Process (MDP) and derive its solution using reinforcement learning (RL) based Neural Fitted *Q*-Iteration (NFQI) algorithm. The reason for choosing RL with neural network function classifier is based on the type of system being modeled and its behavior. As per [38,39], the machine learning algorithms are divided into unsupervised and supervised learning. For unlabeled data algorithms, such as k-means, gaussian mixture models are applied to the data. However, as we have historical data [8] to be used for our modeling, these algorithms will not be the best suited for our scenario. For labeled data training and fitting, algorithms, such as regression, decision trees, support vector machines, naive Bayes classifier and neural networks are used. As the HEMaaS system has user interaction and feedback from wireless access point of the appliances, only using supervised learning algorithms to fit the data for maximum accuracy/minimizing cost will be time and resource consuming. The algorithm has to interact with the environment and objects, learn from their feedback and should update its goals accordingly. Thus reinforcement learning (RL) [40], which starts from a particular state, learns from the environment and update its goals is the best suited for our application. As the algorithm will pass through multiple states in order to reach its optimum goal, a supervised classifier can be used in conjunction with the RL algorithm. Neural network is slow, but classifies accurately in comparison to other supervised learning methods [41]. Hence it is chosen as the modeler for our system.

MDP [42] is a set of discrete time stochastic control process where outcomes are obtained with a combination of partly random events and partly by a decision making process. At each time step, the MDP is modeled as a sequence of finite states $s_{i}\in S$, the agent action $a_{i}\in A$ that are evaluated based on a random process to lead the agent to another state. For each action performed, the agent receives an award *R*. As in [42], MDP is formulated as a set of four-tuple <S,A,P,R>, where *P* is the state transition probability when agent moves from state (s(t)→s(t+1))∈S. From the current state $s_{i}\left(t\right)\in S$ to state $s_{j}\left(t\right)\in S$ in response to action a∈A, the transition probability is $P(s_{i},a,s_{j})$ and an award $R(s_{i},a)$ is received. Let $s_{k}$ denote the state of the system just before the $k^{th}$ transition. In an infinite horizon problem (s→1,…,∞), maximum average discounted reward received is found using the action executed at each state using a reward policy π(s). RL [40] is a machine learning approach that solves the MDP problem. It learns the policy online with real-time interaction with the dynamic environment and adjusts the policy accordingly. After a certain set-up time, the optimal policy can positively be found.

*Q*-learning is an online algorithm that performs reinforcement learning [43]. The algorithm calculates the quality of a state-action pair which is denoted by *Q* and is initialized to zero at the beginning of the learning phase. At each step of environment interaction, the agent observes the environment and decides on an action to change state based on the current state of the system. The new state gives the agent a reward which indicates the value of the state transition. The agent keeps a value function $Q^{\pi }\left(s\left(t\right),a\left(t\right)\right)$ according to an action performed which maximizes the long-term rewards. The *Q*-factor update equation with discounted reward is as follows

(1) $$\begin{array}{c} Q^{t+1}\left(s\left(t+1\right),a\left(t\right)\right)=Q^{t}\left(s\left(t\right),a\left(t\right)\right)+\alpha \left(s\left(t\right),a\left(t\right)\right)[R\left(t\right)+\\ \gamma \cdot MAX\left(Q^{t}\left(s\left(t+1\right),a\left(t\right)\right)\right)-Q^{t}\left(s\left(t\right),a\left(t\right)\right)] \end{array}$$

where, α(s(t),a(t)) is the learning rate (0 <α< 1) and γ is the discount factor within the range 0 and 1. If γ is close to 0, the agent chooses immediate rewards, else it will choose to explore and aim for long-term rewards. In [43] it is proved that the learning rate α is a function of *k*, where *k* is the number of state transitions. It satisfies the condition as

(2) $$\alpha^{k}=\frac{A}{B+k}$$

where, *A* and *B* need to be found out using simulations.

Online learning methods like *Q*-learning are good from a conceptual point of view and are very successful when applied to problems with small, discrete state spaces. However, for more realistic systems, the “exploration overhead”, stochastic approximation inefficiencies and stability issues cause the system to get stuck in sub-optimal policies. Updating the *Q*-value of state-action pair (s(t),a(t)) in time step *t* this may influence the values (s(t−1),a(t)) for all *a*∈*A* of a preceding state $s_{t-1}$. However, this change will not back-propagate immediately to all the involved preceding states. Batch Reinforcement Learning (BRL) typically address all three issues and come up with specific solutions. It performs efficient use of collected historical data and yield better policies [44]. It consists of three phases, which are exploration, learning and application. Exploration has an important impact on the quality of the policies that can be learned. The distribution of transitions in the provided batch must resemble the “true” transition probabilities of the system in order to allow the derivation of good policies. For achieving this, training of samples is done from the system itself, by simply interacting with it. When samples cover the state spaces closed to the goal state, policy achieved will be closed to the optimal policy and convergence would be faster. NFQI algorithm is one of the popular algorithms described in [45]. Given a set of transition samples over (s(t),a(t),R(t),s(t+1)) and an initial *Q*-value ${\bar{q}}_{s,a}^{0}=0$, derive an initial approximation $Q^{0}$ with $Q^{0}={\bar{q}}_{s,a}^{0}$. Update the value of ${\bar{q}}_{s,a}^{k}$ at each iteration. Define a training set $T^{k}$ and convert the update problem into a supervised neural network based learning problem. Finally, find the resulting function approximator ${\hat{Q}}^{i}$ using the pattern trained using set $T^{k}$. At the end, a greedy policy is used to define the policy π(s).

(3) $$\begin{array}{c} \pi \left(s\right)=\underset{a\in A}{argmax}\;Q\left(s,a\right) \end{array}$$

### 3.1. State-Action Modelling of Appliances

The software architecture of the homes in communities shown in Section 2.2 describes a typical condo home architecture with living room, bedroom, kitchen and washroom. Each of the sections have various common home appliances having varied peak load power rating as in Table 2 as taken from [46]. The states s(t) defined in the Algorithm 1 are different combinations of power levels derived from the peak power rating of the appliances. Apart from refrigerator all other appliances can be turned *On/Off* in a smart home as the refrigerator needs to continuously run throughout the day and should not be stopped. Usage pattern of all other appliances vary throughout the day and can be controlled through the MCCU. Therefore, in total there are 10 appliances and a $2^{n}-1$ transition states depicting various combination of power levels (n=9) which results in 511 states. Lets depict each appliance in ascending order of their peak power level from Table 2 with level “$p_{l}$”. Thus Lighting will be symbolized by $p_{1}$ and WasherDryer by $p_{9}$. The power values are coded as binary states i.e., 0 represents the *Off* state and 1 represents *On* state. For example, 001001010 means Microwave, Heater-*2* (Bedroom) and Stove are in *On* state and rest all are in *Off* condition. The total power consumed at that instant *t* is 7600Watts if every *On* appliance is operating at peak load.

**Algorithm 1:** Reward Matrix (R) Computation Algorithm
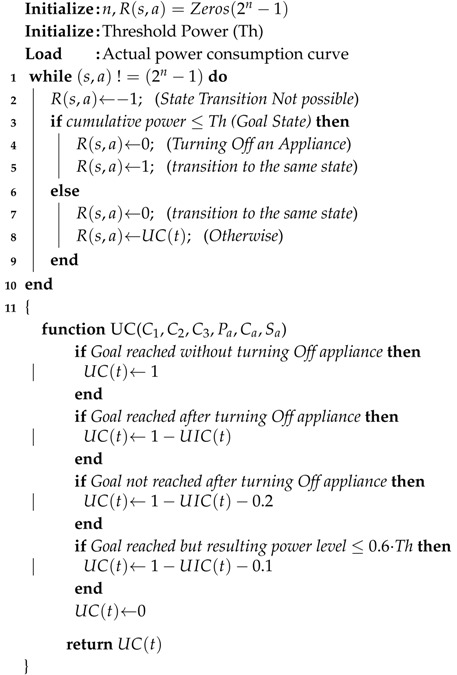


There are four different actions that can be performed based on the states. Turning the appliance *Off*, turning it *On*, pausing the operation and postponing the operation. For the case of simplicity, turning the appliance *Off* is considered to be a required action. Also pausing and postponing the operation of the appliance can be selected for the symbolic *Off* state through the MCCU control based on the situation. The representation remains the same but power level changes. Moreover, we also define User Input Preferences (UIP) as a user input control which changes the decision of the MCCU controller algorithm as desired by the user at a certain time interval. After the scheduling task is over, the control is shifted back to MCCU algorithm. Agent can move from one state to another state after performing an action. The user inconvenience is modeled in the reward matrix and the goal of the strategy is to minimize the user inconvenience.

### 3.2. User Convenience and Reward Matrix

In this section, user convenience UC(t) is modeled at a time instant *t* and the goal is to maximize the UC(t). The reward values for turning off an appliance is based on the user inconvenience. The parameters taken to model UC(t) are user preference $\left(P_{a}\left(t\right)\right)$ of the appliances, power consumption energy cost saving $\left(C_{a}\left(t\right)\right)$, and robustness $\left(S_{a}\left(t\right)\right)$. Maximum inconvenience is caused by turning off an user preffered appliance at a given time. The time slot is discretized for every 15 min regarding the preferences and is divided into four times of the day i.e., Morning (MR), Afternoon (AF), Evening (EV) and Night (NT). Table 3 depicts the $\left(P_{a}\left(t\right)\right)$ values of the appliances for different times of the day and the preferences are set according to a typical winter usage in Canada.

User inconvenience UIC(t) due to turning off an appliance with preference represented by Table 3 will become

(4) $$\begin{array}{c} UIC\left(t\right)=C_{1}\cdot P_{a}\left(t\right) \end{array}$$

$C_{1}$ is a constant and is set to 1 to give user preference maximum importance while choosing the agent action. As appliances are turned off, energy savings in terms of the cost is achieved. So turning *Off* the maximum power consuming appliance at a given time *t* will give the maximum convenience to the users in terms of cost savings. Rest all appliances’ energy cost is normalized w.r.t the maximum power load of the maximum power consuming appliance at *t*. User inconvenience UIC(t) due to turning off an appliance is also dependent on the cost saving $\left(C_{a}\left(t\right)\right)$.

(5) $$\begin{array}{c} UIC\left(t\right)=C_{2}\cdot \left(1-C_{a}\left(t\right)\right) \end{array}$$

The more the cost saving, the lesser the user inconvenience. However, cost cannot be saved sacrificing preference comfort for users. Hence, constant $C_{2}$ will have lower contribution to the UIC(t). We take $C_{2}$ as 0.5 here for our case. Emergency $\left(E_{a}\left(t\right)\right)$ gives users options for choosing to start an appliance regardless of the time of the day, power consumed and preference control. When the user chooses to run an appliance, it becomes a do not care condition in the state for that instant *t*. Hence the number of state-action pair for the reward matrix decreases. The appliance power is subtracted from the goal usage power.

Section 2.2 describes how MQTT handles broker security with Password Authentication, Client ID Authentication, SSL/TLS Certification and firewalls. Robustness of a system shows how it is immune to security threats and fault tolerant. Less robust system also creates inconvenience to the users. Robustness of the system is modelled behaviourly has been categorized as {*Good, Medium and Bad*}. For each behaviour of the system a constant $C_{3}$ value have been assigned to the UIC(t) function as

(6) $$\begin{array}{c} UIC\left(t\right)=C_{3}\cdot S_{a}\left(t\right)\\ C_{3}=\left\{\begin{array}{c} 0.2,Good\\ 0.3,Medium\\ 0.5,Bad \end{array}\right. \end{array}$$

The user experiences more inconvenience for a Bad system as compared to a Good system in terms of their robustness measure. User convenience UC(t) is calculated from Equations (4)–(6) as

(7) $$\begin{array}{c} UC\left(t\right)=1-\left\{\frac{C_{1}P_{a}\left(t\right)+C_{2}\left(1-C_{a}\left(t\right)\right)+C_{3}S_{a}\left(t\right)}{3}\right\} \end{array}$$

Reward matrix (R) is based on the user convenience values for each appliance using Algorithm 1. Size of the reward matrix depends on the number of appliances and the number of power levels the house agent can occupy. The size of the reward matrix for this problem is 255×255. Power level zero is not taken into consideration as it is impossible for the power to reduce to zero level in a home throughout the day. Algorithm 1 depicts the steps to formulate the reward matrix and is true for any number of state transitions. According to required threshold power (Th) to be achieved, reward matrix R(s,a) is computed as per Algorithm 1. Th is the goal state where the optimization of power stops. The goal state may be reached with or without turning off an appliance. According to the power level where the goal state is reached, user convenience value is penalized. The most penalty is for goal state not being reached even after turning off an appliance. The penalties are 0.1 at goal state power less than or at 60% of threshold power and 0.2 for goal not being reached even after turning off an appliance.

## 4. NFQbHEM

The proposed Neural Fitted *Q*-based Home Energy Management (*NFQbHEM*) algorithm is described in this section. The algorithm is based on the RL-based NFQI method as in Section 3. The algorithm works in three phases: exploration, training and application. In the exploration phase, *NFQbHEM* captures the historical demand data based on different seasons [46]. Winter month data has been chosen in our application. The algorithm is defined in Algorithm 2 and the steps are listed as follows:
**Algorithm 2:**
*NFQbHEM* Algorithm
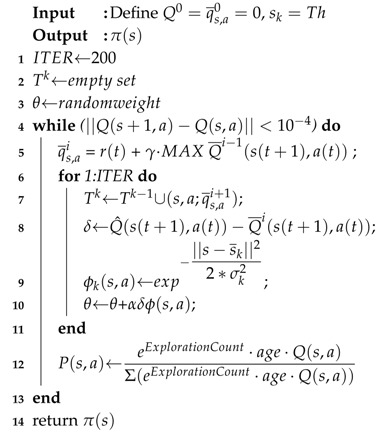


**Exploration Phase**:

*Step 0 (Inputs)*: Set the *Q*-factors to some arbitrary values (e.g., 0).
*Step 1*: For each state *s*, the set of admissible actions, *a* is defined, and an action a∈A is chosen randomly and applied. After applying a(t) in s(t), the next state s(t+1) is reached and the immediate reward r(t) from Algorithm 1 is calculated.
*Step 2*: The set of (s(t),a(t),R(t),s(t+1)) is inserted from the environment as a new sample *F*. Repeating the process, sufficient samples are found to train the algorithm.

**Training Phase:**

*Step 1*: The training initializes $Q^{0}={\bar{q}}_{s,a}^{0}=0$, and tries to find a function approximator ${\hat{Q}}^{i}$.
*Step 2*: Similar to the *Q*-update process, append a corresponding pattern set $T^{k}$ to the set $\left(s,a;{\bar{q}}_{s,a}^{i+1}\right)$.
*Step 3*: As our historical data is a curve fitting problem, Radial Basis Function Neural Network (RBFNN) [47] is chosen to approximate the function Q(s,a).
*Step 4*: The feature function ϕ: *S* x *A* maps each state-action pair to a vector of feature values.
*Step 5*: θ is the weight vector specifying the contribution of each feature across all state-action pairs. The weight is updated at each iteration. The training is done for 200 iterations in our case.

**Execution Phase:**

*Step 1*: Current data determine the state of the system.
*Step 2*: A greedy policy is used to find the policy π(s) as in Equation (3).
*Step 3*: Later in learning with more episodes, exploitation makes more sense because, with experience, the agent can be more confident about what it knows.
*Step 4*: Stopping criterian with absolute error
  $$||Q\left(s+1,a\right)-Q\left(s,a\right)||<10^{-4}.$$

## 5. Experimental Results

**Figure 3:**
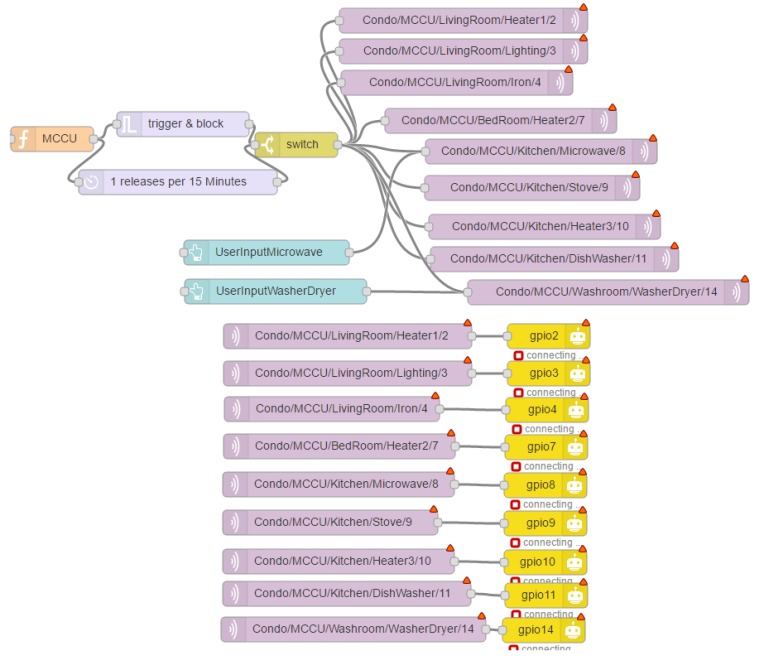
User interface design.

**Figure 4:**
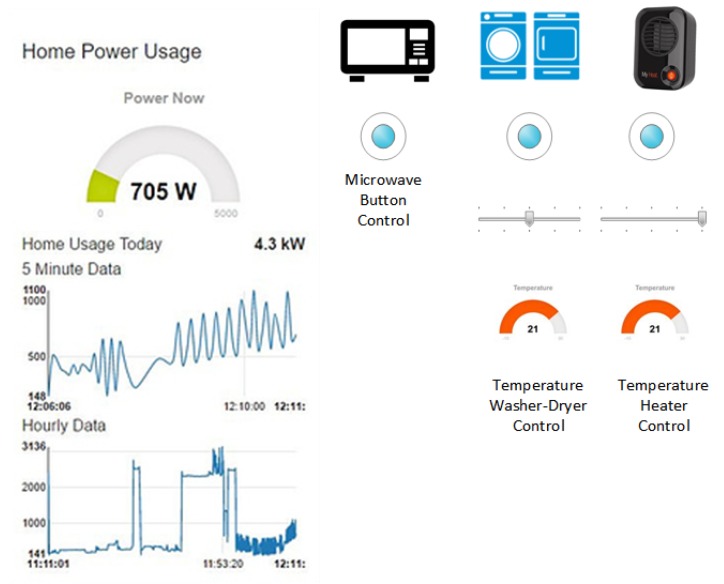
HEM Interface.

This section describes the simulated results of HEMaaS platform with the *NFQbHEM* algorithm to control 10 appliances in a sample condo home in a smart community. Due to the experimental nature of the setup and results, the results have been presented in the context of the sample condo home. Comparison with other architectures in literature have not been drawn as the method described here is unique to the setup and it would be unfair to compare algorithms with different setup. Due to hardware complexity, it is very hard to implement other algorithms for the setup explained in this work. As explained in the software architecture and communication interface in Section 2.2, the MCCU consists of a Raspberry-Pi3 deploying a node-red platform. MQTT (Mosquitto) is used as the broker between the MCCU publisher and subscribing home appliances. Custom python code with the *NFQbHEM* algorithm deployed on it runs on the Raspberry-Pi3 to control the home appliances’ *Delay/Pause/On/Off* operation via sonoff wi-fi switches through a MQTT gateway. The node-red dashboard interface designed in this work offers an easy and convenient user interface (UI) for a homeowner to interact with the HEMaaS system. Figure 3 and Figure 4 illustrates our user interface (UI) flow design and dashboard control respectively. The UI shows the node-RED flow of the different appliances as connected to the MCCU. Each appliance is controlled through a GPIO pin and follows the Home/(Room)/(Device)/Pin hierarchy. The proposed UI also offers several visualization features to a user. They can have access to real-time and historical appliance usage information with graphs via the sonoff accumulated data information of real time power usage. User Input preferences (UIP) can be set via the dashboard. The different options which are available include setting a temperature for Heater-1, Heater-2, Heater-3 and washer-dryer, rescheduling washer-dryer operation and starting necessary appliances immediately bypassing the automated control for a particular duration. The UI is also accessible from anywhere in the world via the smart-phone app. If for any reason there is a communication failure, the local settings of the appliances will take precedence.

Matplot library of python gives us the tool to analyse the power demand data for different cases. Two different cases have been discussed here for analyzing and plotting our results.

**Case I**: A sample day’s total power consumption data is compared with different peak power reduction of 5%,10%,15% and 20% of the total peak demand. The user convenience is also shown as a comparison.
**Case II**: The user convenience in terms of random (*Good, medium and bad*) behavior of the system is analyzed in this case.

For the Case I above, the energy in KWh savings and reduction in carbon-footprint for a community consisting of 85 condos of our typical architecture as in Section 2.1 is also plotted.

### 5.1. Case I

**Figure 5:**
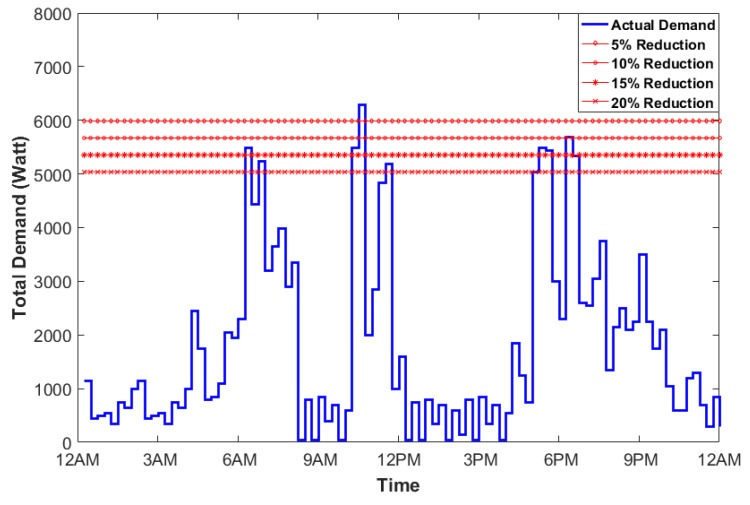
Plot of the total demand versus time during a typical Canadian winter month in Ontario.

In this section, the actual power consumption plot is generated using 10 smart appliances. The plot in Figure 5 shows the peak demand in watts versus time of the day. The interval of time duration is 15 min. Starting with initial Q(s,a), the HEMaaS platform has to learn to find the optimal path when peak demand power during a certain interval exceeds the available power. The available power is taken as a percentage reduction of the peak power. 5%,10%,15% and 20% are taken as the peak reduction percentages to test and validate our methodology. Algorithm 2 has been initialized with starting parameters of learning α=0.5, discount γ=0.8, *A* and *B* as 90 and 100 respectively. The center state ${\bar{s}}_{k}$ is taken as the median power consumed at a particular interval. The peak power is 6300 watts and Algorithm 1 depicts the reward matrix initial computation. The total energy consumption historical data of a typical condo has been taken from national resources canada [48] for a typical winter month in canadian ontario province. The feature function ϕ is derived from approximating the curve of the historical data and is used to train the weight vector θ. When the total power consumption is greater than the peak power power reduction, it selects actions (randomly) and moves from current state to a new state, receives reward and then it starts issuing control signals (Delay/Pause/On/Off) to other appliances until one of the goal states is reached.

**Figure 6:**
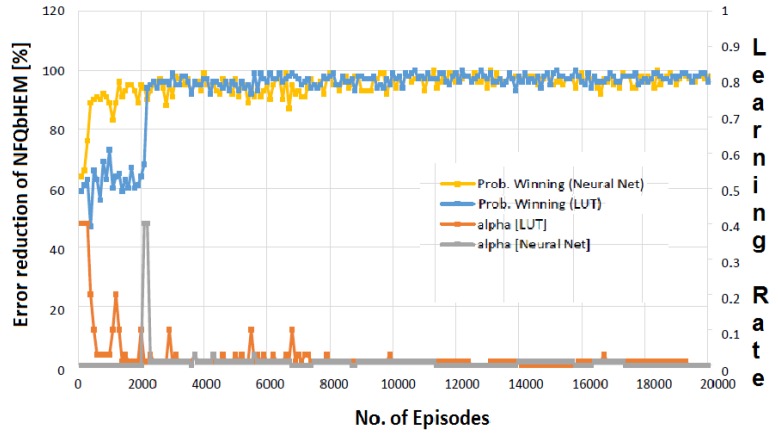
Plot of sample episodic run NFQbHEM learning process.

**Figure 7:**
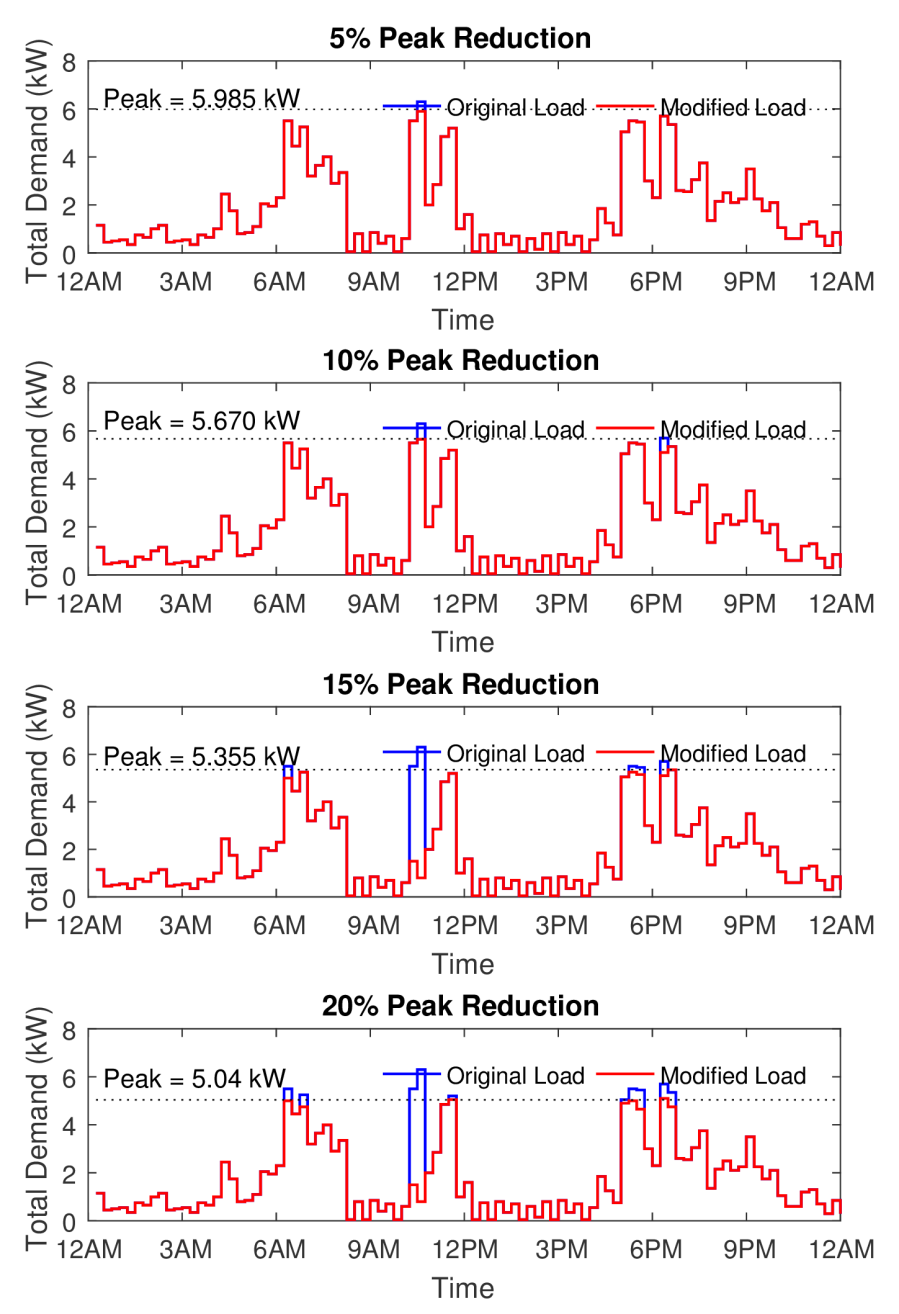
Plot of the total demand versus time for different peak reduction percentages.

Figure 6 depicts the learning process of the NFQbHEM algorithm. The graph is plotted between number of episodes algorithm running for look-up table based *Q*-learning and the neural network *Q*-learning-based NFQbHEM algorithm. The proposed algorithm learns faster and reaches a stable value in only about 570 episodes as compared to look-up only based *Q*-learning. The algorithm is stopped at 570 episodes as the error achieved is $10^{-5}$. Thus neural network modeler helps the *Q*-learning achieve its goal state faster. Figure 7 shows the total demand versus time for different peak reduction percentages. Once the optimal policy is found, the MCCU will execute the sequence of rules (turning off appliances, rescheduling their timing of operation and temperature control one by one) until the goal state with maximum user convenience is reached. At the optimal policy, MCCU determines when the power goes above the desired reduction, it modifies its power as in Figure 7. Table 4 shows the appropriate actions taken by the MCCU unit at varied time intervals for different appliances.

**Figure 8:**
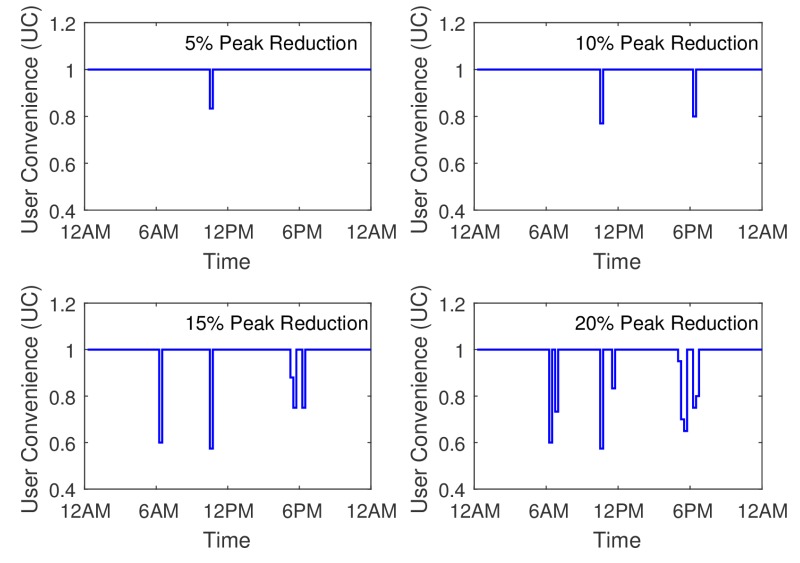
Plot of the user convenience (uc) versus time.

The user convenience (UC), is shown in Figure 8 for the four peak reduction threshold values. It can be inferred from the figures that the UC decreases with the increase in the threshold for power saving. Some of the peak load consumption which lies during the afternoon and evening time slots are affected severely. One suggestion of improvement in the user convenience could be having a variable thresholds for the NFQbHEM algorithm. Therefore the times of day having maximum user utility power consumption, the available power threshold can be increased and can be compensated with a lower available power threshold during other Off peak times while maintaining the overall average power threshold at the same level. If the user convenience level can be maintained more than 70% for most times of the day, then the HEMaaS system can be successful in delivering a coherent and inter-operable platform.

**Figure 9:**
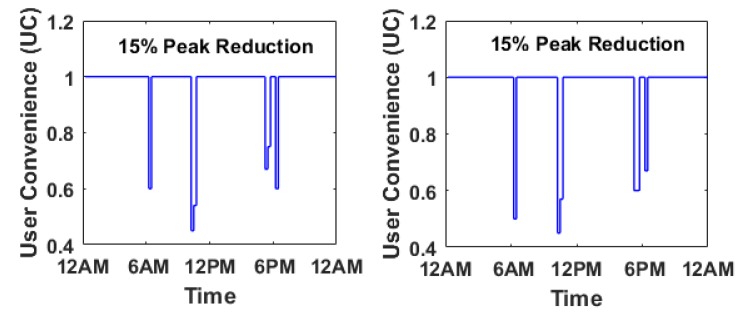
Plot of the user convenience (uc) versus time for (20% Good, 60% Medium and 20% Bad) and (10% Good, 40% Medium and 50% Bad) robustness measure.

In this section, the behavioral modeling of the system is considered in terms its robustness. Robustness measure evaluates a systems quality in terms of security and fault tolerance. To simulate this behavior in the system, the UC(t) from Equation (6) has been chosen with randomly assigning measure of robustness $C_{3}$ at different time intervals. The power level of peak reduction at 15% is taken as the threshold. Figure 9 depicts the UC w.r.t the time of the day and is compared for two different situations. The first situation has (20% Good, 60% Medium and 20% Bad) robustness measure and the second situation has (10% Good, 40% Medium and 50% Bad) robustness measure respectively. The user convenience is severely affected for both cases specifically in the second situation, due to presence of more faulty/malicious channel. Thus security and fault tolerance is shown to have significant effect to the users. Once the UC goes below 50%, the system is considered to be a very poorly managed system where users are forced to save energy sacrificing their comfort, which is highly undesirable.

### 5.2. Case II

**Figure 10:**
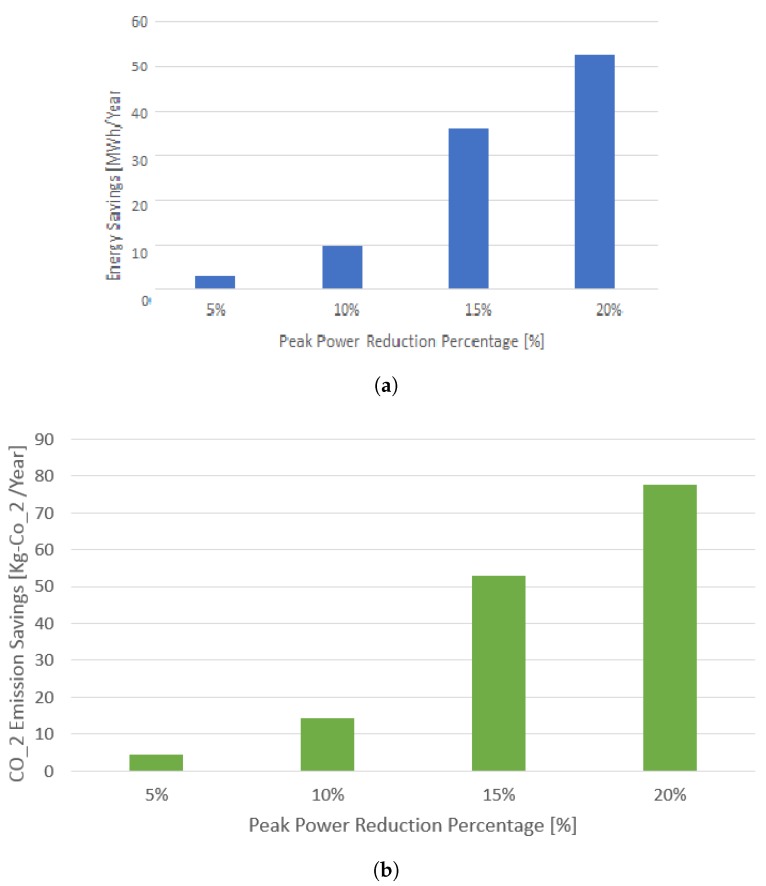
Comparison of peak reduction energy savings and carbon-footprint reductions. (**a**) Energy savings with peak demand reduction; (**b**) Plot of carbon-footprint reduction.

The carbon intensity per KWh (CIPK) is a fundamental measure of a sustainable society. The lesser the CIPK, the better the society in terms of its environment and livability index. The energy savings that are obtained from results in Section 5.1 can be seen as potential price saving for the community as well as a means of reducing the $CO_{2}$ gas emissions. As Canada is progressing towards a sustainable green building infrastructure, it is a healthy sacrifice to have some inconvenience to achieve the greater benefit of having a greener environment in terms of achieving lesser carbon emissions. From [48], the Ontario province’s CIPK is obtained as 125 $gr-CO_{2}$ per KWh. Using the CIPK, the energy savings and carbon emission savings have been computed for a community consisting of 85 condos. Figure 10 shows the energy savings in Mega-Watt-hour (MWh) per year. It also shows the carbon-footprint savings in $Kg-CO_{2}$ per year. The improvement is nearly 14 times from 5% to 15% peak power reduction, which is quite substantial.

## 6. Conclusions

Energy management in smart cities is an indispensable challenge to address due to rapid urbanization. In this paper, we first present an overview of energy management in smart homes to build a green and sustainable smart city, and then present a unifying framework for IoT in building green smart homes. To achieve our goal, a neural network based *Q*-learning algorithm is proposed to reduce the peak load demand of a typical Canadian home while minimizing the user inconvenience and enhancing the robustness of the system. The user convenience level for 5% and 10% load reduction is maintained at and above 80%. Whereas other levels of peak power reduction causes more discomfort for the users. While Canada Green Building Council is working towards finding ways of making buildings greener and community sustainable, a novel method has been applied for finding suitable strategies for efficient management of home energy demand and reducing the energy consumption during peak period in a typical Canadian Condo. In a purely market-driven energy management environment, peak-reduction is mostly valuable for utilities. In order to make the demand side management more user friendly and consumer centric, a reward matrix-based self-learning algorithm has been applied. The energy savings and carbon-footprint reduction is also shown to be quite significant. In future, it has been planned to incorporate real time scheduling into the system to schedule and pause appliance operation. Moreover, it is also proposed to design a system that learns from feedback smart sensors in the environment to ease the MCCU decision making and reduce user input, yet still maintaining a high enough user convenience.

## Figures and Tables

**Table 1:** Nomenclature.

Abbreviations	Expanded Form
IoT	Internet of Things
DR	Demand Response
DSM	Demand side Management
TOU	Time-of-Use
HEMaaS	Home Energy Management as a Service
RL	Reinforcement Learning
MDP	Markov Decision Process
NFQbHEM	Neural Fitted *Q*-based Home Energy Management
MCCU	Main Command and Control Unit
CCM	Community Cloud Management
NAT	Network Address Translation
MQTT	Message Queue Telemetry Transport
UI	User Interface
NFQI	Neural Fitted *Q*-Iteration
UIP	User Input Preferences
R	Reward Matrix
UC	User Convenience
CIPK	Carbon intensity per Kilo-Watt-hour
MWh	Mega Watt-hour

**Table 2:** Maximum Load Rating of Home Appliances.

Appliances	Peak Power Rating [Watts]
Heater-1 (Living Room)	2500
Heater-2 (Bedroom)	2000
Heater-3 (Kitchen)	1500
Iron Center	1000
Microwave	1100
Dishwasher	1300
Lighting	600
Stove	5000
Washer Dryer	5500
Refrigerator	150

**Table 3:** User Preference of Appliances ($P_{a}$).

Appliances	Morning (MR)	Afternoon (AF)	Evening (EV)	Night (NT)
Heater-1 (Living Room)	1	0.3	1	0.3
Heater-2 (Bedroom)	1	0.3	0.4	1
Heater-3 (Kitchen)	0.6	0.3	0.7	0.1
Iron Center	0.6	0.1	0.1	0.1
Microwave	1	0.1	0.8	0.1
Dishwasher	0.5	1	0.3	0.7
Lighting	0.4	0.1	0.7	0.1
Stove	0.7	0.1	1	0.1
Washer Dryer	0.6	0.6	0.3	0.5

**Table 4:** Actions Taken by MCCU.

Time	Required Load Reduction	Required Action
**5% Reduction Threshold**		
10:15–10:30 a.m.	400 W	Turn off the Heater-1 and Heater-3
**10% Reduction Threshold**		
10:15–10:30 a.m.	650 W	Turn off the Heater-1, Heater-2 and Heater-3
6:00–6:15 p.m.	600 W	Reduce the temp. setting of Heater-1
**15% Reduction Threshold**		
6:00–6:15 a.m.	500 W	Reduce the temp. setting of Heater-1 and Heater-2
10:00–10:15 a.m.	1500 W	The temperature setting of the washer-dryer may be changed to reduce the power demand or washer-dryer operation may be rescheduled to another time.
10:15–10:30 a.m.	1500 W	The temperature setting of the washer-dryer may be changed to reduce the power demand or washer-dryer operation may be rescheduled to another time.
5:00–5:15 p.m.	250 W	Turn off the Heater-1
5:15–5:30 p.m.	300 W	Turn off the Heater-2
6:00–6:15 p.m.	600 W	Reduce the temp. setting of Heater-1
**20% Reduction Threshold**		
6:00–6:15 a.m.	500 W	Reduce the temp. setting of Heater-1 and Heter-2
6:30–6:45 a.m.	500 W	Turn off the Heater-2
10:00–10:15 a.m.	1500 W	The temperature setting of the washer-dryer may be changed to reduce the power demand or washer-dryer operation may be rescheduled to another time.
10:15–10:30 a.m.	1500 W	The temperature setting of the washer-dryer may be changed to reduce the power demand or washer-dryer operation may be rescheduled to another time.
11:15–11:30 a.m.	150 W	Refrigerator Turned Off
4:45–5:00 p.m.	150 W	Turn off the Refrigerator
5:15–5:30 p.m.	500 W	Turn off the Heater-3
5:30–5:45 p.m.	800 W	Turn off the Heater-2 and Heater-3
6:00–6:30 p.m.	600 W	Reduce the temp. setting of Heater-1

## References

[1] Zanella A., Bui N., Castellani A., Vangelista L., Zorzi M. (2014). Internet of Things for Smart Cities. IEEE Intern. Things J..

[2] Klein C., Kaefer G. (2008). From smart homes to smart cities: Opportunities and challenges from an industrial perspective. Proceedings of the International Conference on Next Generation Wired/Wireless Networking, St. Petersburg, Russia, 3–5 September 2008.

[3] Sheng Z., Yang S., Yu Y., Vasilakos A.V., Mccann J.A., Leung K.K. (2013). A survey on the ietf protocol suite for the internet of things: Standards, challenges, and opportunities. IEEE Wirel. Commun..

[4] Liao C.F., Chen P.Y. (2017). ROSA: Resource-Oriented Service Management Schemes for Web of Things in a Smart Home. Sensors.

[5] Mendes T.D., Godina R., Rodrigues E.M., Matias J.C., Catalão J.P. (2015). Smart home communication technologies and applications: Wireless protocol assessment for home area network resources. Energies.

[6] Ejaz W., Naeem M., Shahid A., Anpalagan A., Jo M. (2017). Efficient Energy Management for the Internet of Things in Smart Cities. IEEE Commun. Mag..

[7] Hsu Y.L., Chou P.H., Chang H.C., Lin S.L., Yang S.C., Su H.Y., Chang C.C., Cheng Y.S., Kuo Y.C. (2017). Design and Implementation of a Smart Home System Using Multisensor Data Fusion Technology. Sensors.

[8] Daily T. (2016). Survey of Commercial and Institutional Energy Use, 2014.

[9] Canada Green Building Council. https://www.cagbc.org/.

[10] DMO S. (2016). Smart Home Report.

[11] Kani S.A.P., Nehrir M.H. (2013). Real-time central demand response for primary frequency regulation in microgrids. IEEE Trans. Smart Grid.

[12] Borenstein S., Jaske M., Rosenfeld A. (2002). Dynamic Pricing, Advanced Metering, and Demand Response in Electricity Markets.

[13] Palensky P., Dietrich D. (2011). Demand side management: Demand response, intelligent energy systems, and smart loads. IEEE Trans. Ind. Inform..

[14] Rodrigues E.M., Godina R., Shafie-khah M., Catalão J.P. (2017). Experimental Results on a Wireless Wattmeter Device for the Integration in Home Energy Management Systems. Energies.

[15] Zhou B., Li W., Chan K.W., Cao Y., Kuang Y., Liu X., Wang X. (2016). Smart home energy management systems: Concept, configurations, and scheduling strategies. Renew. Sustain. Energy Rev..

[16] Díaz Pardo de Vera D., Siguenza Izquierdo A., Bernat Vercher J., Hernández Gómez L.A. (2014). A Ubiquitous Sensor Network Platform for Integrating Smart Devices into the Semantic Sensor Web. Sensors.

[17] Farhangi H. (2010). The path of the smart grid. IEEE Power Energy Mag..

[18] Kumar A., Hancke G. (2014). An Energy-Efficient Smart Comfort Sensing System Based on the IEEE 1451 Standard for Green Buildings. IEEE Sens. J..

[19] Shen V.R., Yang C.Y., Chen C.H. (2015). A smart home management system with hierarchical behavior suggestion and recovery mechanism. Comput. Stand. Interfaces.

[20] Rahman M., Kuzlu M., Pipattanasomporn M., Rahman S. Architecture of web services interface for a Home Energy Management system. Proceedings of the 2014 IEEE PES, Innovative Smart Grid Technologies Conference (ISGT).

[21] Lee Y.T., Hsiao W.H., Huang C.M., Seng-cho T.C. (2016). An integrated cloud-based smart home management system with community hierarchy. IEEE Trans. Consum. Electron..

[22] Ciancetta F., D’Apice B., Gallo D., Landi C. (2007). Plug-n-Play Smart Sensor Based on Web Service. IEEE Sens. J..

[23] Chen C., Wang J., Heo Y., Kishore S. (2013). MPC-based appliance scheduling for residential building energy management controller. IEEE Trans. Smart Grid.

[24] Li S., Zhang D., Roget A.B., O’Neill Z. (2014). Integrating home energy simulation and dynamic electricity price for demand response study. IEEE Trans. Smart Grid.

[25] Wei Q., Lewis F.L., Shi G., Song R. (2017). Error-Tolerant Iterative Adaptive Dynamic Programming for Optimal Renewable Home Energy Scheduling and Battery Management. IEEE Trans. Ind. Electron..

[26] Mohsenian-Rad A.H., Wong V.W., Jatskevich J., Schober R., Leon-Garcia A. (2010). Autonomous demand-side management based on game-theoretic energy consumption scheduling for the future smart grid. IEEE Trans. Smart Grid.

[27] Dehghanpour K., Nehrir H., Sheppard J., Kelly N. (2016). Agent-based modeling of retail electrical energy markets with demand response. IEEE Trans. Smart Grid.

[28] Magno M., Polonelli T., Benini L., Popovici E. (2015). A low cost, highly scalable wireless sensor network solution to achieve smart LED light control for green buildings. IEEE Sens. J..

[29] O’Neill D., Levorato M., Goldsmith A., Mitra U. Residential Demand Response Using Reinforcement Learning. Proceedings of the First IEEE International Conference on Smart Grid Communications.

[30] Ruelens F., Claessens B.J., Vandael S., Iacovella S., Vingerhoets P., Belmans R. Demand response of a heterogeneous cluster of electric water heaters using batch reinforcement learning. Proceedings of the Power Systems Computation Conference.

[31] Turitsyn K., Backhaus S., Ananyev M., Chertkov M. Smart finite state devices: A modeling framework for demand response technologies. Proceedings of the 50th IEEE Conference on Decision and Control and European Control Conference.

[32] Kara E.C., Berges M., Krogh B., Kar S. Using smart devices for system-level management and control in the smart grid: A reinforcement learning framework. Proceedings of the IEEE Third International Conference on Smart Grid Communications (SmartGridComm).

[33] Node-RED. https://nodered.org/.

[34] Sonoff Pow WiFi Switch with Power Consumption Measurement. https://www.itead.cc/sonoff-pow.html.

[35] Raspberry Pi 3 Model B https://www.raspberrypi.org/products/raspberry-pi-3-model-b/.

[36] Hunkeler U., Truong H.L., Stanford-Clark A. MQTT-S: A publish/subscribe protocol for Wireless Sensor Networks. Proceedings of the 3rd International Conference on Communication Systems Software and Middleware and Workshops, COMSWARE.

[37] An Open Source MQTT v3.1/v3.1.1 Broker. https://mosquitto.org/.

[38] Bishop C.M. (2006). Pattern Recognition and Machine Learning.

[39] Pedregosa F., Varoquaux G., Gramfort A., Michel V., Thirion B., Grisel O., Blondel M., Prettenhofer P., Weiss R., Dubourg V. (2011). Scikit-learn: Machine learning in Python. J. Mach. Learn. Res..

[40] Sutton R.S., Barto A.G. (1998). Reinforcement Learning: An Introduction.

[41] Andrew A.M., Zakaria A., Mad Saad S., Md Shakaff A.Y. (2016). Multi-Stage Feature Selection Based Intelligent Classifier for Classification of Incipient Stage Fire in Building. Sensors.

[42] Puterman M.L. (2014). Markov Decision Processes: Discrete Stochastic Dynamic Programming.

[43] Watkins C.J., Dayan P. (1992). Q-learning. Mach. Learn..

[44] Ernst D., Geurts P., Wehenkel L. (2005). Tree-based batch mode reinforcement learning. J. Mach. Learn. Res..

[45] Riedmiller M. Neural fitted Q iteration–first experiences with a data efficient neural reinforcement learning method. Proceedings of the European Conference on Machine Learning.

[46] Natural Resources Canada (2012). Energy Consumption of Major Household Appliances Shipped in Canada, Summary Report.

[47] Park J., Sandberg I.W. (1991). Universal approximation using radial-basis-function networks. Neural Comput..

[48] IESO Ontario Power Stats—Canadian Energy Issues. http://canadianenergyissues.com/ontario-power-stats/.

